# Changes in female sexual function during the retirement transition

**DOI:** 10.1097/GME.0000000000002778

**Published:** 2026-04-07

**Authors:** Viivi Virkkunen, Katja Kero, Sari Stenholm, Päivi Polo-Kantola

**Affiliations:** 1 Department of Obstetrics and Gynecology, Turku University Hospital, University of Turku, Turku, Finland; 2 Department of Public Health, University of Turku, Turku University Hospital, Turku, Finland; 3 Centre for Population Health Research, University of Turku, Turku University Hospital, Turku, Finland; 4 Research Services, Turku University Hospital, University of Turku, Turku, Finland

**Keywords:** Aging, Retirement, Sexual dysfunction, Sexual health, Woman

## Abstract

**Objectives:**

For many women, retirement represents a significant transition in late adulthood, which may result in many alterations in lifestyle habits and behavioral patterns. Several factors known to improve during the retirement transition are also associated with female sexual function; however, the literature on the influence of retirement on female sexual function remains limited. In this study, we examined changes in female sexual function during the retirement transition.

**Methods:**

The population for this prospective follow-up study consisted of 110 women (mean age 63.1 y) from the Finnish Retirement and Aging study cohort. Data were collected annually before and after retirement. Sexual function was assessed with the Female Sexual Function Index questionnaire, which includes a total score and subdomain scores for desire, arousal, lubrication, orgasm, pain, and satisfaction.

**Results:**

The total Female Sexual Function Index score remained stable from preretirement to postretirement. The desire score improved from the preretirement level by 0.11 points (95% CI: 0.01-0.20, *P*=0.03), whereas the lubrication score changed by −0.29 points, although this change did not reach statistical significance (95% CI: −0.60 to 0.03, *P*=0.07). The scores for arousal, orgasm, pain, and satisfaction remained stable.

**Conclusions:**

During the retirement transition, sexual function remained mostly unchanged, but sexual desire improved. Future studies with longer follow-ups and larger sample sizes are warranted to investigate the evolution of female sexual function after retirement and identify the potential underlying factors of this phenomenon.

The prevalence of female sexual dysfunction (FSD) increases with age.^1^ As the underlying factors of this phenomenon are multidimensional; considerable variation has been reported in sexual function among older women.^2-6^ Poor physical^1^ and mental health, as well as work-related stress, have been shown to negatively affect female sexual function.^4,7,8^ In addition, relationship satisfaction, intimate communication, and the presence of erectile dysfunction in the partner are known to influence women’s sexual function.^3,9^

Menopause is recognized as having an overall negative impact on female sexual function,^5,6^ but the effect of aging after menopause is less clear. Only a handful of longitudinal studies have been conducted on the evolution of female sexual function in postmenopausal women. For example, in an Australian 10-year follow-up study, sexual distress among late postmenopausal women was investigated. Sexual distress, which was measured using the Female Sexual Distress Scale, did not increase among sexually active women, whereas the frequency of sexual activity decreased, and partner-related difficulties increased.^4^ The authors also reported that Female Sexual Function Index (FSFI) scores declined when considering all women, including sexually inactive ones, but remained stable for women maintaining sexual activity.^4^ In another longitudinal cohort study, which was carried out in the UK, the authors found that one in five postmenopausal women developed FSD according to the FSFI during the four-year follow-up period, although 7% of the women reported improved sexual functioning.^2^ Overall sexual functioning according to the FSFI remained relatively stable during the follow-up period in all study participants.^2^ In a Finnish cohort, Katainen and colleagues examined climacteric symptoms among women aged 47-65 years using the Women’s Health Questionnaire, which includes three questions about sexual function.^10^ They reported that the majority of the women discontinued their sexual activity. However, no significant change was found in sexual function among sexually active women during the 19-year follow-up period.^11^

Retirement is an important milestone in late adulthood, and it may result in many changes in lifestyle habits and behavioral patterns.^12^ Although the loss of work-related activities and changes in social connections and roles may pose challenges to retirees, scholars have recently suggested that retirement generally has a favorable influence on health.^13,14^ During the retirement transition, psychological distress has been found to decrease,^15^ whereas sleep duration increases and sleep difficulties lessen.^16-18^ In addition, recent studies have shown an increase in leisure-time physical activity after retirement.^13,14,19^ These factors, among others, could mediate the observed improvements in self-reported physical and mental health,^20-22^ as well as life satisfaction,^23^ that occur after retirement.

Despite these changes during the retirement transition, the literature on the evolution of female sexual function in this period is insufficient. To the best of our knowledge, only one longitudinal study with a 4-year follow-up has been published on sexual satisfaction as an indicator of sexual function during retirement.^24^ In this study, both partnered men and women were included, and the retirement status of the partner was considered as a confounding factor.^24^ The results showed no changes in female sexual satisfaction after retirement. Still, women who presented higher sexual satisfaction before retirement exhibited a notable decrease afterward.^24^ Importantly, no studies have been conducted on changes in other aspects of female sexual function during the retirement transition, such as sexual desire, arousal, orgasm, or pain. To address this gap in the literature, we examined changes in female sexual function during the retirement transition, focusing on both overall sexual function and its subdomains.

## METHODS

### Participants

**FIG. 1:**
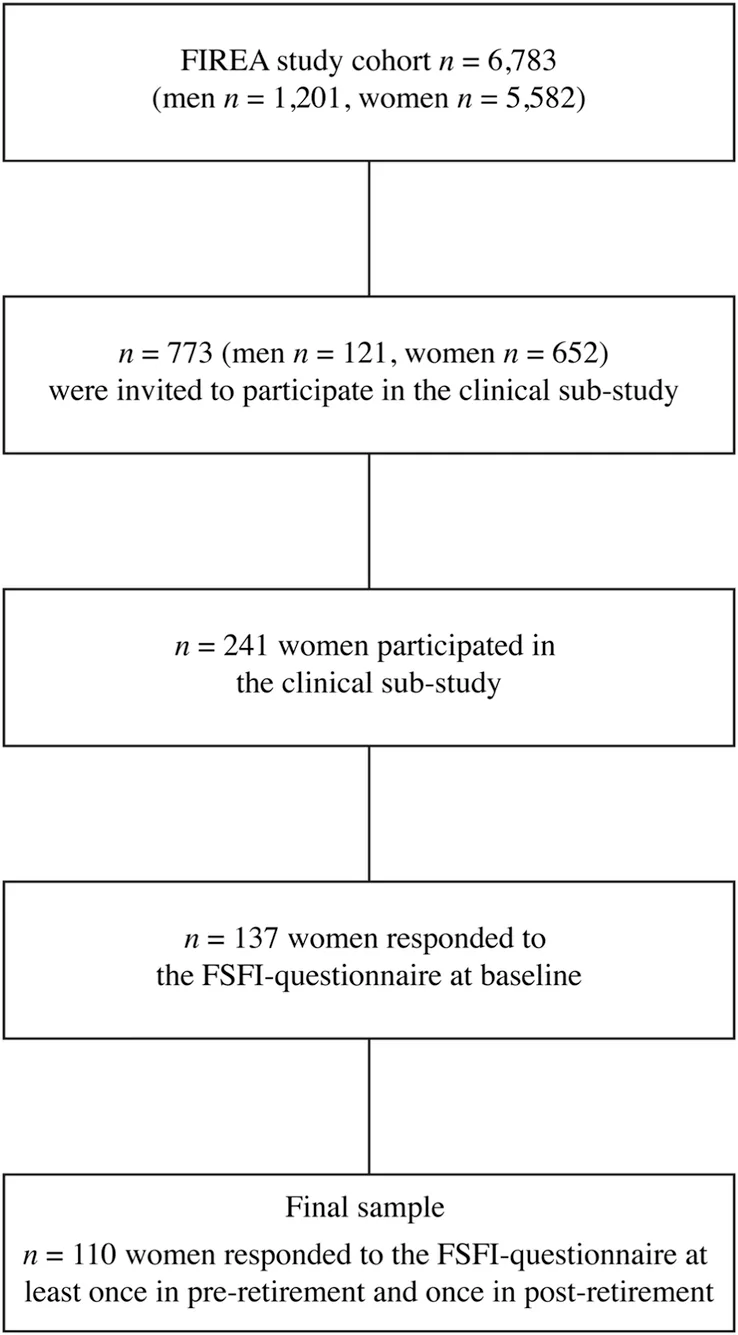
Flowchart of the study. FIREA, Finnish Retirement and Aging Study; FSFI, Female Sexual Function Index.

This research was conducted as part of the Finnish Retirement and Aging (FIREA) study, an ongoing prospective cohort study of public sector employees in Finland established in 2013. A detailed description of the FIREA study design and implementation has been provided elsewhere.^2^ For the present research, we included women who participated in a clinical substudy (n=241) and responded to the FSFI questionnaire (n=137) at the study baseline, while they were still working. Participants were then followed up with annual measurements until they retired. To determine the timing of retirement, the working status was collected during the annual clinical visit. The data were subsequently centered around the retirement transition and categorized as preretirement (waves −2 to −1), retirement transition (waves −1 to +1), and postretirement (waves +1 to +2). To be included in the analysis, responses to the FSFI questionnaire were required at least once preretirement and once postretirement. This resulted in a study sample of 110 participants (Fig. 1).

### Ethical approval

The FIREA study was conducted in accordance with the Helsinki Declaration and approved by the Ethics Committee of the Hospital District of Southwest Finland (ETMK: 84/1801/2014). All the participants provided written informed consent.

### Finland’s retirement system

In Finland, public sector employees may retire on a statutory basis after the age of 63 years, and at the latest, before the age of 68 years. Each individual born in a certain year has their own retirement age, which is tied to their life expectancy. Postponing retirement from the estimated retirement age increases one’s pension-income level. After the pension reform of 2017, employees are able to retire on a partial early statutory retirement age pension, a years-of-service pension (for those who have worked for at least 38 years in a job that requires great mental or physical effort), or a full or partial disability pension.^25^ Because of the inclusion criteria, the FIREA study participants were mostly retiring on a statutory basis, with a mean age of 64.0 years (SD: 1.4).^26^

### Assessment of sexual function

The FSFI questionnaire is used to evaluate sexual function.^27^ It assesses sexual function over the preceding 4 weeks, regardless of relationship status. It includes 19 questions, which cover the following six subdomains: desire (two questions), arousal (four questions), lubrication (four questions), orgasm (three questions), satisfaction (three questions), and pain (three questions). Sexual function is assessed based on the total score and the subdomain scores. Total scores range from 2 to 36, with higher scores indicating better sexual function. Regarding the subdomains, desire and satisfaction have minimum scores of 1, which indicate low desire and dissatisfaction, respectively. Arousal, lubrication, orgasm, and pain have minimum scores of 0, which indicate that the respondent had no sexual activity. The maximum score for each subdomain is 6.

The FSFI subdomain score was calculated for each domain that had at least one question answered. For those respondents with one or more missing values in the domain-specific questions, the mean value across the questions was used for that domain. The total scores were calculated for all the questionnaires in which at least three subdomain questions were completely or partially answered.

### Confounding factors

The participants’ dates of birth and preretirement occupational titles were obtained from the Keva Public Sector Pensions Register. The women were divided into two occupational status groups according to their occupational titles before retirement and based on the International Standard Classification of Occupations (ISCO). The two groups were manual workers (eg, cleaners and maintenance workers; ISCO classes 5-9) and nonmanual workers (eg, teachers, physicians, registered nurses, and technicians; ISCO classes 1-4).^28^ Population density at the participants’ residential locations was used as a proxy for the degree of urbanization. Information about the number of inhabitants within a 250 × 250 m square was obtained from the Statistics Finland database.^29^ Areas with fewer than 200 inhabitants per 250 × 250 m were coded as low density, whereas areas exceeding this threshold were categorized as high density. Marital status was surveyed and dichotomized as single/married or cohabiting. Women self-reported any chronic illnesses diagnosed by a physician. Menopausal symptoms, including vasomotor symptoms (hot flashes, sweating) and vaginal dryness, were surveyed, and participants categorized as having or not having any symptoms. Information on the use of menopausal hormone therapy (MHT) (yes/no) and/or local estrogen (yes/no) was collected, and the data were combined as one variable for the analyses. Smoking (nonsmoker [never or former/current smoker]) and alcohol consumption (beer, wine, and/or spirits, with problematic alcohol use defined as >16 drinks per week)^30^ were self-reported. The Beck Depression Inventory was used to evaluate depressive symptoms.^31^ Obesity was defined as having a body mass index (BMI) ≥ 30 kg/m^2^. Low physical activity was defined as physical activity of <14 metabolic equivalent hours per week.^32^

### Statistical analyses

Characteristics of the study population in the last measurement before retirement are shown as percentages for categorical variables and as means and SDs for continuous variables.

Linear regression analyses with generalized estimating equations (GEEs) were used (proc genmode in SAS) to estimate the mean levels and 95% CIs of the FSFI total and subdomain scores in the preretirement years (study waves −2 and −1) and postretirement years (study waves +1 and +2). As repeated measurements were used, the GEE model controlled for the intraindividual correlation between repeated measurements. The model uses an exchangeable correlation structure and is not sensitive to measurements missing completely at random.^33,34^ Changes in the FSFI total and subdomain scores during the retirement transition were estimated based on all available preretirement and postretirement measurements by using contrast statements in SAS, and the results were calculated as mean changes and their 95% CIs. The changes in the FSFI total and subdomain scores were first examined in the whole population, and additional sensitivity analysis was carried out only with the participants who reported being sexually active. All analyses were adjusted for age, occupation, marital status, and use of MHT and/or local estrogen. These covariates were selected a priori because they have been shown to be associated with female sexual function^3,35^ and/or retirement timing.^36^

All statistical analyses were performed using SAS version 9.4 (SAS Institute Inc., Cary, NC).

## RESULTS

The characteristics of the study participants before retirement (study wave −1) are shown in Table 1. The mean age was 63.1 years (SD: 1.1). About two-thirds (73%) of the women worked in nonmanual occupations, and the majority were married or cohabiting (75%). Most of the participants (77%) lived in low population density residential areas. Only three participants changed their status from unmarried to married or cohabiting during the retirement transition. Of all the women, 41% reported menopausal symptoms, 33% used systemic MHT, and 53% employed local estrogen treatment. Also, 30% of the women reported low physical activity, and 16% were obese. Only 5% of the participants reported current smoking or problematic alcohol use. Chronic illnesses in the study population are shown in Table 1. Overall, the women were relatively healthy. Regarding this aspect, 6% had a cardiovascular disease; 5% had diabetes; 61% reported having osteoarthritis, and 11% scored over 10 points in the Beck Depression Inventory, indicating at least mild depressive symptoms. Compared with the FIREA survey and clinical substudy female participants, the present study’s participants were more often married or cohabiting, less often obese, and less frequently depressed (Table S1, Supplemental Digital Content 1, http://links.lww.com/MENO/B505).

**TABLE 1:** Characteristics of the study participants before retirement (study wave −1) (n=110)

	Mean (SD)	Range
Age, y	63.1(1.1)	60-66
	n (%)	
Occupational status		
Manual	30 (27.3)	
Nonmanual	80 (72.7)	
Marital status		
Single	26 (25)	
Married or cohabiting	78 (75)	
Missing	6	
Population density in the residential neighborhood		
Low	77 (71.3)	
High	31 (28.7)	
Missing	2	
Systemic menopausal hormone therapy		
Yes	34 (33.3)	
No	68 (66.7)	
Missing	8	
Local estrogen		
Yes	54 (52.9)	
No	48 (47.1)	
Missing	8	
Local lubricant		
Yes	22 (21.6)	
No	80 (78.4)	
Missing	8	
Menopause symptoms		
Yes	42 (41.2)	
No	60 (58.8)	
Missing	8	
Lifestyle-related risk factors		
Smoking	5 (4.7)	
Problematic alcohol use	5 (4.7)	
Low physical activity	30 (28.6)	
Obesity^*a*^	16 (14.6)	
Chronic diseases		
Cardiovascular disease^*b*^	6 (6.1)	
Diabetes	5 (5.1)	
Osteoarthritis	62 (60.8)	
Depression^*c*^	12 (10.8)	
Suboptimal self-rated health	21 (19.8)	

^*a*^ Defined as body mass index ≥ 30 kg/m^2^.

^*b*^ Defined as angina pectoris, myocardial infarction, stroke, and/or claudication.

^*c*^ Beck Depression Inventory score > 10 points.

**FIG. 2:**
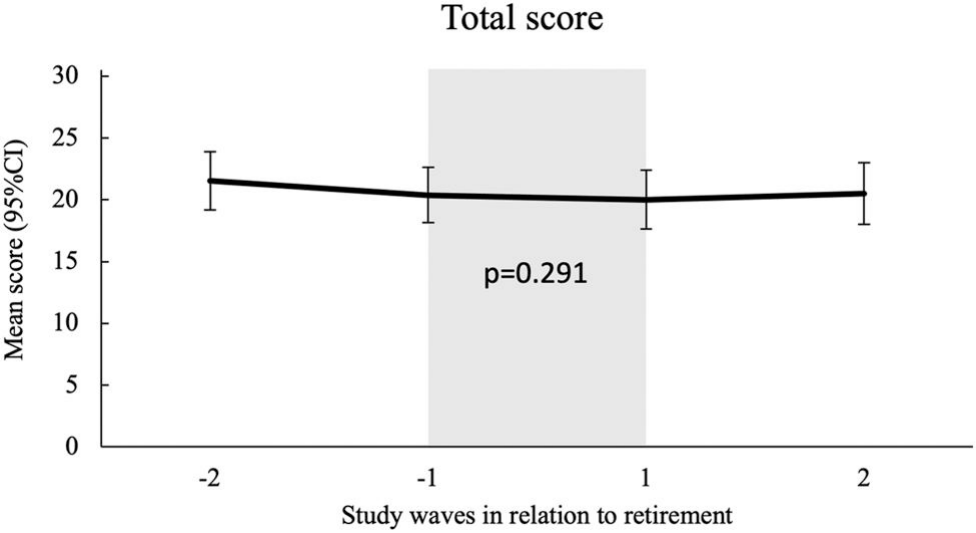
FSFI total score during the retirement transition (study waves −2 to +2) (n=110). FSFI, Female Sexual Function Index.

**FIG. 3:**
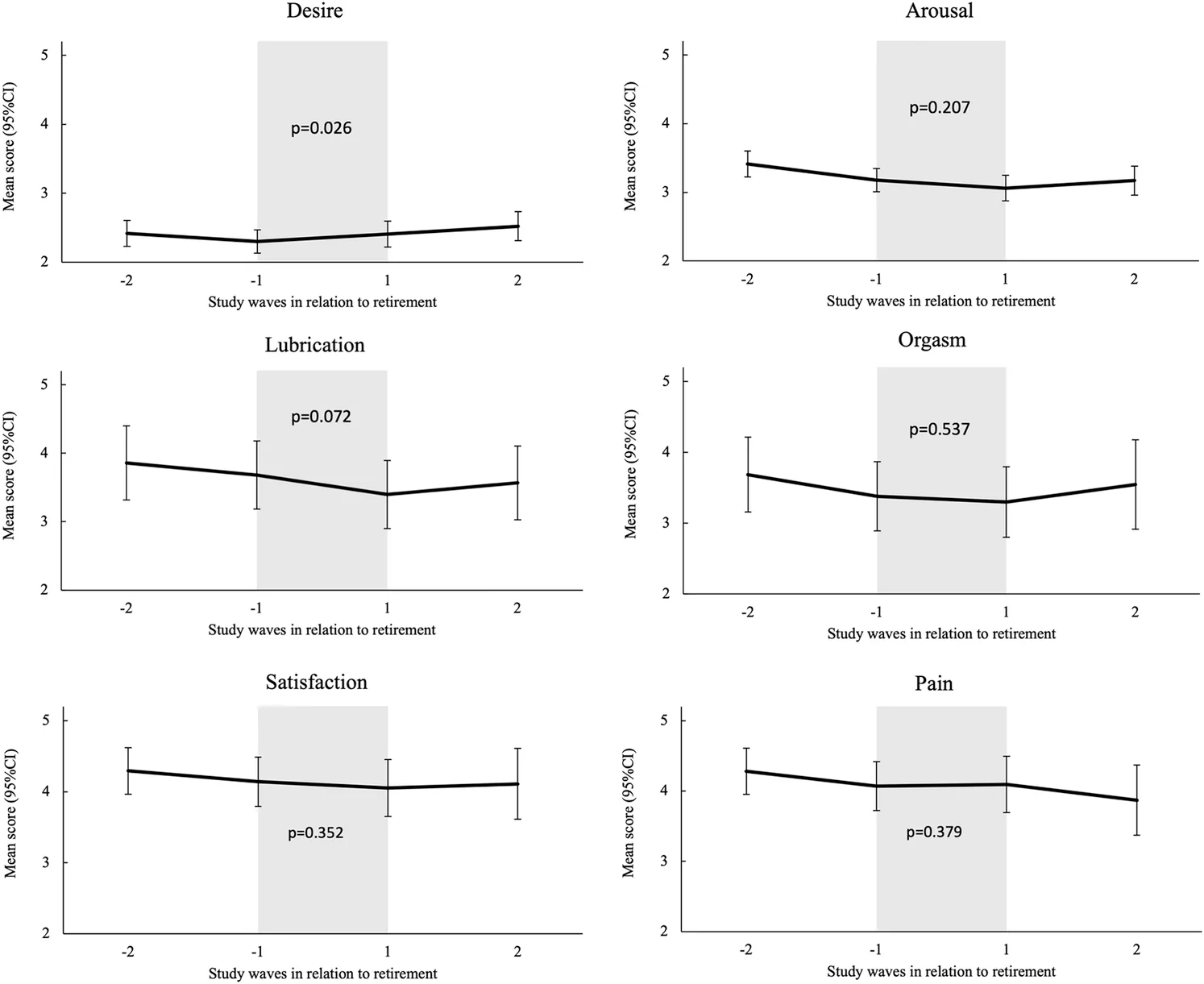
FSFI subdomain scores during the retirement transition (study waves −2 to +2) (n=110). FSFI, Female Sexual Function Index.

Figure 2 presents the FSFI total scores before and after retirement. In the study wave before retirement, the mean FSFI total score was 20.4 points (95% CI: 18.1-22.6). The total score remained stable during the retirement transition, without a statistically significant change (mean: −0.71, 95% CI: −2.02 to 0.61). Figure 3 shows the FSFI subdomain scores across the retirement transition. Of the FSFI subdomains, the highest mean scores before retirement were in satisfaction (mean: 4.1, 95% CI: 3.7-4.5) and pain (mean: 4.1, 95% CI: 3.6-4.5), which indicates high sexual satisfaction and low experienced pain during sexual activity. The lowest mean scores were in desire (mean: 2.3, 95% CI: 2.1-2.5) and arousal (mean: 3.2, 95% CI: 2.7-3.6), which indicates reduced functioning in these areas. During the retirement transition, the desire subdomain score increased from preretirement to postretirement by 0.11 points (95% CI: 0.01-0.20). Furthermore, the lubrication score showed a borderline statistically significant decrease from preretirement to postretirement of 0.29 points (95% CI: −0.60 to 0.03). No changes were observed in the arousal, orgasm, satisfaction, and pain subdomains scores during the retirement transition.

Figures S1 and S2, Supplemental Digital Content 1, http://links.lww.com/MENO/B505 show the changes in the FSFI total and subdomain scores during the retirement transition among the women who reported being sexually active. The mean FSFI total score before retirement was 24.1 points (95% CI: 22.5-25.7), and it remained stable during the retirement transition (mean: −0.80, 95% CI: −2.17-0.57). The desire score increased by 0.11 points (95% CI: 0.02-0.21) from preretirement to postretirement. Moreover, the lubrication score decreased by 0.37 points (95% CI: −0.70 to −0.04) from preretirement to postretirement, whereas the arousal, orgasm, satisfaction, and pain subdomain scores showed no changes during the retirement transition.

## DISCUSSION

To the best of our knowledge, this is the first prospective study to examine changes in different aspects of female sexual function in relation to the retirement transition. We found that sexual desire improved, and lubrication decreased during this transition; however, the changes were minor, and overall sexual function remained quite unchanged throughout the period in question.

Our findings complement and broaden the results reported by Henning et al^24^ in a Swedish follow-up study of concentrating on sexual satisfaction in men and women during the retirement transition. Henning et al’s^24^ study demonstrated that sexual satisfaction decreased in men, whereas it remained stable in women during and after retirement. In our study, we found that overall sexual function remained stable during the retirement transition, as was the case for sexual satisfaction. The results of a few recent longitudinal studies show that sexual function remains unchanged among sexually active aging women.^4,11^ Although we focused on investigating the period around retirement, our findings align with previous research on aging women.

In our study, we examined changes in different subdomains of female sexual function during the retirement transition, which is a novel aspect. Contrary to the improvement in the desire subdomain, lubrication tended to decrease from preretirement to postretirement. Furthermore, when investigating lubrication only in the sexually active women, the detected decrease reached statistical significance. Lubrication has been shown to diminish at the beginning of menopause due to a reduction in estrogen levels.^37^ Systemic MHT and local estrogen are commonly used to alleviate menopausal symptoms, including vaginal dryness. These treatments have been shown to positively impact sexual function.^38^ Although the use of systemic MHT or local estrogen in relation to retirement has not been previously studied, it is possible that these therapies, especially systemic MHT, are discontinued after retirement, as menopausal symptoms may interfere less with daily life once women have stopped working. Importantly, even though systemic menopausal symptoms may ease over time, vulvovaginal dryness often continues to progress.^37,39^ The majority of the women in our study used systemic MHT and/or local estrogen both before and after retirement. However, we did not assess the doses they used; therefore, at least in some women, the doses might have been too low to maintain vaginal health, leading to a decrease in lubrication. As for the menopausal state, we did not have information on the age of menopause. Still, it can be assumed that all our participants were postmenopausal as the mean age of our study population was 63.1 years (range: 60-66), and the mean age for menopause in Finland is 51 years.^40^

One of the major strengths of our study is the use of the FSFI questionnaire, a validated and widely used instrument to evaluate FSD.^27^ In addition, we accounted for several confounding factors known to be associated with female sexual function. Moreover, all the women were about the same age and retired at the statutory age; this means that younger participants who retired prematurely due to disability or those who started part-time retirement were excluded from the study. Only three participants reported a change in their relationship status, which did not compromise the results. Furthermore, the use of systemic MHT and local estrogen in our study population aligns with the previously reported use of MHT in postmenopausal women in Finland, which makes our findings generalizable to the Finnish population.^41^

We included in the analyses all the women who responded to the FSFI questionnaire at least once before and after retirement, regardless of their reported sexual activity. We did not assess sexual behavior or habits specifically, as these aspects could have been too sensitive and personal, and they probably would have led to inadequate reporting of sexual activity or even withdrawal from the study. Also, it is possible that the women who had worse sexual function could have felt that this issue was too personal to discuss. Conservative attitudes toward sexuality might be associated with greater FSD.^42^ Furthermore, the term “sexual activity” can be interpreted differently in the age group in question. We did not specifically survey all the different forms of sexual behavior women engage in, such as oral sex or masturbation. However, we conducted additional analyses only with sexually active women. The changes observed in the entire study population and the sexually active women were similar, except in regard to lubrication, which declined during the retirement transition in the sexually active women.

Our study also has a few limitations that need to be addressed. First, the study population was relatively small, which may have reduced the study’s statistical power. The present study was conducted as part of the clinical substudy of the FIREA cohort study, and women participating voluntarily were asked to answer the FSFI questionnaire. Completion of this questionnaire was optional, which may have contributed to a low response rate. The study population was highly selected, as it consisted of women working in the public sector, able to retire on a statutory basis, and willing to participate in a study regarding sexual function. These inclusion criteria most likely restricted the generalizability of the results. Furthermore, fewer women were obese and had depression in the present study population compared with the FIREA survey cohort and clinical substudy female participants; thus, our participants might have been healthier compared with women of the same age in Finland.

Second, our follow-up time was four years, with the retirement date set in the middle. Previously, Lintuaho et al^43^ reported that improvements in self-perceived health and increases in physical activity lasted only for a short period after retirement. On the basis of our data, we cannot comment on how female sexual function would progress over time after retirement. Thus, future studies with longer follow-ups are warranted.

Third, we did not use the Female Sexual Distress Scale in our assessment of female sexual function. The *Journal of Sexual Medicine* recently published a study suggesting the use of this scale in combination with the FSFI questionnaire to assess whether sexual impairments are accompanied by distress.^44^ According to FSD clinical assessment criteria, sexual complaints are not considered FSD without associated distress.^45^ Therefore, we could not estimate the prevalence or magnitude of FSD in the study population.

## CONCLUSIONS

Our results show that retirement induced no considerable change in sexual function in women, with sexual function remaining stable during the retirement transition. Minor variations were observed in some subdomains, with sexual desire improving and lubrication tending to decrease. In the West, more people are aging and thus entering retirement; for these people, the quality of life, which includes sexual function, is essential. Studies with longer follow-up periods, as well as larger and more diverse samples, are needed to better understand the progression of female sexual function during and after retirement and gain more knowledge of the potential underlying factors of this phenomenon.
